# Spatio-temporal trends of malaria incidence from 2011 to 2017 and environmental predictors of malaria transmission in Myanmar

**DOI:** 10.1186/s40249-023-01055-6

**Published:** 2023-01-28

**Authors:** Yan Zhao, Pyae Linn Aung, Shishao Ruan, Kyawt Mon Win, Zifang Wu, Than Naing Soe, Myat Thu Soe, Yaming Cao, Jetsumon Sattabongkot, Myat Phone Kyaw, Liwang Cui, Lynette Menezes, Daniel M. Parker

**Affiliations:** 1 Department of Immunology, College of Basic Medical Sciences, China Medical University, Shenyang, 110122 Liaoning China; 2 Myanmar Health Network Organization, Yangon, Myanmar; 3 Mahidol Vivax Research Unit, Faculty of Tropical Medicine, Mahidol University, Bangkok, Thailand; 4 Department of Public Health, Ministry of Health, NayPyiTaw, Myanmar; 5 Division of Infectious Diseases and International Medicine, Department of Internal Medicine, Morsani College of Medicine, University of South Florida, 3720 Spectrum Boulevard, Suite 304, Tampa, FL 33612 USA; 6 Department of Population Health and Disease Prevention, Department of Epidemiology, University of California, Irvine, USA

**Keywords:** Spatial distribution, Temporal clustering, Spatiotemporal clustering, Environmental predictor, *Plasmodium falciparum*, *Plasmodium vivax*, Myanmar

## Abstract

**Background:**

Myanmar bears the heaviest malaria burden in the Greater Mekong Subregion (GMS). This study assessed the spatio-temporal dynamics and environmental predictors of *Plasmodium falciparum* and *Plasmodium vivax* malaria in Myanmar.

**Methods:**

Monthly reports of malaria cases at primary health centers during 2011–2017 were analyzed to describe malaria distribution across Myanmar at the township and state/region levels by spatial autocorrelation (Moran index) and spatio-temporal clustering. Negative binomial generalized additive models identified environmental predictors for falciparum and vivax malaria, respectively.

**Results:**

From 2011 to 2017, there was an apparent reduction in malaria incidence in Myanmar. Malaria incidence peaked in June each year. There were significant spatial autocorrelation and clustering with extreme spatial heterogeneity in malaria cases and test positivity across the nation (*P* < 0.05). Areas with higher malaria incidence were concentrated along international borders. Primary clusters of *P. falciparum* persisted in western townships, while clusters of *P. vivax* shifted geographically over the study period. The primary cluster was detected from January 2011 to December 2013 and covered two states (Sagaing and Kachin). Annual malaria incidence was highest in townships with a mean elevation of 500‒600 m and a high variance in elevation (states with both high and low elevation). There was an apparent linear relationship between the mean normalized difference vegetative index and annual *P. falciparum* incidence (*P* < 0.05).

**Conclusion:**

The decreasing trends reflect the significant achievement of malaria control efforts in Myanmar. Prioritizing the allocation of resources to high-risk areas identified in this study can achieve effective disease control.

**Graphical Abstract:**

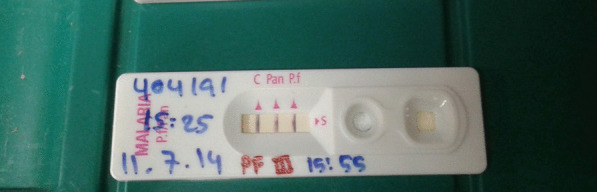

**Supplementary Information:**

The online version contains supplementary material available at 10.1186/s40249-023-01055-6.

## Background

The global malaria burden is still enormous; there were about 247 million cases in 2021, of which 5.4 million cases occurred in Southeast Asia [1]. The Greater Mekong Subregion (GMS) consists of Cambodia, Laos, Myanmar, Thailand, Vietnam, and two provinces of China (Yunan and Guangxi), where Myanmar (87.7%) accounted for most of the indigenous cases of malaria in 2021 [1]. Myanmar accounted for most of the *Plasmodium falciparum* malaria cases within the region. As falciparum malaria cases declined over time, *P. vivax* has become the dominant species [2]. Despite the overall decline of malaria incidence over the last several years, Myanmar still reports the majority of malaria cases and deaths in the GMS [3–5].

The Mekong Malaria Elimination (MME) program is an initiative to support the GMS countries in achieving the goal of malaria elimination by 2030. However, there are several potential barriers to eliminating malaria in the GMS. First, the emergence and spread of parasites resistant to antimalarial drugs and mosquitoes resistant to insecticides hinder the elimination course. Previous reports of asymptomatic *P. falciparum* and *P. vivax* isolates carrying genes potentially associated with drug resistance suggest a silent spread of drug-resistant parasites in Myanmar [6–8]. The vector species display tremendous spatial heterogeneity in distribution in the GMS, many of which have developed resistance to insecticides used in public health interventions [9–11]. Second, there is a trend for higher levels of transmission and malaria-related mortality near international borders, making ‘border malaria’ a concern for malaria prevention. Third, because of internal military conflicts in Myanmar, refugees and internally displaced people rushed to and settled down along the international borders, increasing the risk of infectious diseases. Border regions frequently exhibit cultural and linguistic heterogeneity, complicating healthcare education, disease prevention, and administration.

Monitoring border malaria is important, but an overall analysis at the national level is essential for governments or organizations to rationally allocate material and human resources for malaria control. By 2016, 291 out of 330 townships in Myanmar were endemic, with more than 40 million people at risk for malaria [12]. According to the data on malaria cases from October to December 2021, *P. falciparum* and *P. vivax* cases were distributed in the western, southern and northeast regions of Myanmar, whereas the central regions were essentially devoid of malaria [2]. This spatial heterogeneity, or uneven distribution of malaria cases across the landscape, occurs at multiple spatial scales. Some nations have a greater burden than others, some states or provinces within nations have greater burdens than others, and communities likewise vary in their malaria burdens. Spatial analyses that describe and help explain the spatial and spatio-temporal patterns of malaria are important for public health surveillance, implementation of interventions, and for general policies [13–15]. For monthly cases, May to August each year is the peak season of malaria infection in Myanmar [2]. However, the temporal trends of *P. falciparum* and *P. vivax* malaria in Myanmar are unclear.

Thus, the present study explored the spatiotemporal dynamics of malaria incidence in Myanmar, at both township and state/region levels, to obtain a granular view of the heterogeneous malaria distribution, detect malaria clusters for targeted control, and identify environmental predictors of *P. falciparum* and *P. vivax* malaria. Such information will be essential to informing regional malaria elimination efforts.

## Methods

### Study area

Myanmar lies between latitudes 9° and 29° N and longitudes 92° and 102° E; has a total area of 678,500 km^2^; and is bordered by China, Laos, Thailand, Bangladesh and India. The nation is divided into five physiographic regions: the northern mountains, the western ranges, the eastern plateau, the central basin and lowlands, and the coastal plains. There are three seasons: the cool season (late October to mid-February), the hot-dry season (mid-February to mid-May), and the rainy season (mid-May to late October). Agriculture, forestry, and fishing constitute the most significant contributors to Myanmar’s economy. Annual rainfall in the delta region is approximately 2500 mm, while the average annual rainfall in the dry zone in central Myanmar is less than 1000 mm [16]. Myanmar has seven states and seven regions, including 63 districts and 330 townships, with a total population of about 50,279,900 people in the 2014 census.

### Data source

Data for this study were obtained from the National Malaria Control Program (NMCP) and other partners such as the Myanmar Medical Association, Medical Action Myanmar, Myanmar Council of Churches, and Myanmar Health Assistant Association. The data include yearly counts of malaria cases and tests at the township level from 2011 to 2017 and monthly malaria cases at the state or regional level from 2011 to 2016. Population counts likewise came from the NMCP. Townships are smaller administrative units than states and regions. Areas defined as either states or regions have been classified based on political and socio-cultural factors but at the same administrative level.

Environmental predictor variables for vegetation and surface flooding were derived from Moderate Resolution Imaging Spectroradiometer (MODIS) products (MOD13Q1/MYD13Q1 250 m AQUA/TERRA 16-day composites). Since many infectious diseases, especially vector-borne diseases, are strongly influenced by environmental factors, we hypothesized that indicators of vegetation and surface flooding would correlate with malaria cases. Three environmental indices were downloaded and considered in these analyses: the normalized difference vegetation index (NDVI), the enhanced vegetation index (EVI), and a normalized flooding index (NFI) [17]. NFI is indicative of surface water, NDVI is indicative of green surface vegetation, and EVI is an improved measure of green vegetation that is intended to account for dense forest canopies and atmospheric conditions that can lead to errors in NDVI measurements. Data were downloaded for each of these environmental indices (EI) within each 16-day period from 2011 to 2017. NDVI and EVI were strongly colinear, so we retained only EVI as an indicator of vegetation in our models. These variables were summarized at the township level by calculating the mean NFI and mean EVI for each year and for each township.

Elevation data were also downloaded from the Shuttle Radar Topography Mission 30 m dataset (https://srtm.csi.cgiar.org/srtmdata/), accessed through DIVA-GIS (https://www.diva-gis.org/gdata). Summary statistics (mean elevation and variance in elevation) were calculated for each administrative unit using QGIS 3.10.1 software (Open Source Geospatial Foundation, https://www.qgis.org).

### Dynamics of malaria incidence and test positivity

Two primary epidemiological metrics were used in this research: the annual parasite incidence (API, also referred to as the case incidence) and the test positivity. API is the ratio of the number of symptomatic clinical cases of malaria, microscopically or rapid diagnostic test (RDT) confirmed, in a population in a given year to the total population of the region in that year (reported as the number of cases per 1000 people). Test positivity is the number of positive cases divided by the total number of tests. We used both in this research because testing intensity can influence case incidence. For example, some places with little-to-no testing may have high malaria burdens that are not obvious when mapping or otherwise presenting case incidence alone. Malaria cases from the two most prevalent human *Plasmodium* parasites (*P. falciparum* and *P. vivax*) were also shown separately.

We plotted API and test positivity by species, across years for the entire nation, monthly for the entire nation, and annually by state or region. National and regional malaria API and test positivity for *P. falciparum* and *P. vivax* were calculated by year and graphed to show annual fluctuations. Species incidences for each month during 2011–2016 were calculated to observe seasonal fluctuations in malaria transmission [18].

We also mapped estimated crude API, smoothed API, and test positivity at the township level. Empirical Bayes smoothing (SEB) was used with the API estimates to account for potential variance instability from differences in population estimates across geographic units (which can lead to spurious outliers that appear to be hotspots or coldspots of disease). SEB smoothing improves the ability to identify overarching spatial patterns in diseases and other phenomena [19, 20]. These smoothed APIs were calculated using GeoDa 1.14.0 software (Open Source, https://geodacenter.github.io), all maps were generated using QGIS version 3.10.1 (https://www.qgis.org).

### Spatial patterns in API and test positivity

We used three approaches for analyzing spatial autocorrelation of API and test positivity: spatial correlograms, the Moran’s *I* statistic (both global and local), and scan statistics. Spatial correlograms illustrate the magnitude of spatial autocorrelation that exists (on the y-axis, with 1 indicating perfect clustering, − 1 indicating perfect dispersal, and 0 indicating no spatial pattern) between pairs of administrative units at different distances away from each other (distances along the x-axis).

We then tested for global and local spatial autocorrelation using Moran’s *I* statistic and local indicator of spatial autocorrelation (LISA). Moran’s *I* ranges from 1 to − 1, with a score of zero indicating the null hypothesis of spatial randomness. Positive values indicate clusters of malaria cases, while negative values indicate that neighboring areas are characterized by different malaria cases (i.e. areas with high cases neighboring areas with low cases). The LISA statistics were investigated and mapped to identify four types of clusters (high-high, low-low, high-low, and low-high) of smoothed *P. falciparum* and *P. vivax* incidence at the township level. High-high and low-low clusters present hotspots and coldspots, respectively. High-low and low-high categories represent outliers [21]. The statistical significance was tested using 999 Monte Carlo permutations, and a *P*-value of 0.05 or less was considered statistically significant [22]. Both Moran’s *I* and LISA statistics are based on adjacency matrices, and the spatial weight matrix is based on a Queen adjacency matrix, which establishes connections between all neighbors that share a common point or length on the boundary. Moran’s *I* and LISA statistics were calculated using GeoDa 1.14.0 (https://geodacenter.github.io).

Finally, we used Kulldorf’s retrospective space-time scan statistics to test for likely clusters of cases in space and time [23]. Briefly, the process involves scanning data windows across space and time and recording the number of observations and expectations within the window for each location in comparison to expected observations outside of the window. The risk of malaria within and outside the window was tested using a likelihood ratio test with the null hypothesis of equal risk across space. The window with the largest log likelihood ratio (LLR,the most likely cluster) was considered the cluster with the highest malaria risk. The window with the next to maximum LLR represented the secondary likely cluster and was considered the area with the second highest malaria risk. The most likely cluster (primary cluster) was identified based on the maximum log likelihood ratio, other clusters with statistically significant log likelihood values were defined as secondary clusters [24]. We used the discrete space-time Poisson model to look for malaria clusters in space and time, with 1-month temporal aggregations. The scan statistics were calculated using SaTScan™ version 9.3 (Kuldorff M. and Information Management Services, Inc. https://www.satscan.org/).

### Negative binomial regressions for predictors of reported *P. falciparum* and *P. vivax* malaria cases

We used negative binomial generalized additive models to look for predictors of malaria cases at the township level and annually for *P. falciparum* and *P. vivax* malaria, respectively. The models control for repeated measures within administrative units using a random intercept, and for the relative geographic locations of the administrative units using a smoothed spline function interaction term for the geographic locations of the mean centers of the administrative units. Rather than assuming the shape of the potential association between a given variable and the outcome (counts of malaria cases), smoothing splines were fit to the continuous covariates. Covariates in the models included: mean elevation, variance in elevation, mean annual EVI, mean annual NFI, the number of exams in a given location, and the year. Regression analyses were performed separately for falciparum and vivax malaria. The model outputs are presented as plots of the spline functions. The generalized additive negative binomial regressions were calculated using the statistical software R 4.0 (R Core Team, R Foundation for statistical computing, Vienna, Austria).

## Results

### Trends of countrywide malaria incidence

**Fig. 1:**
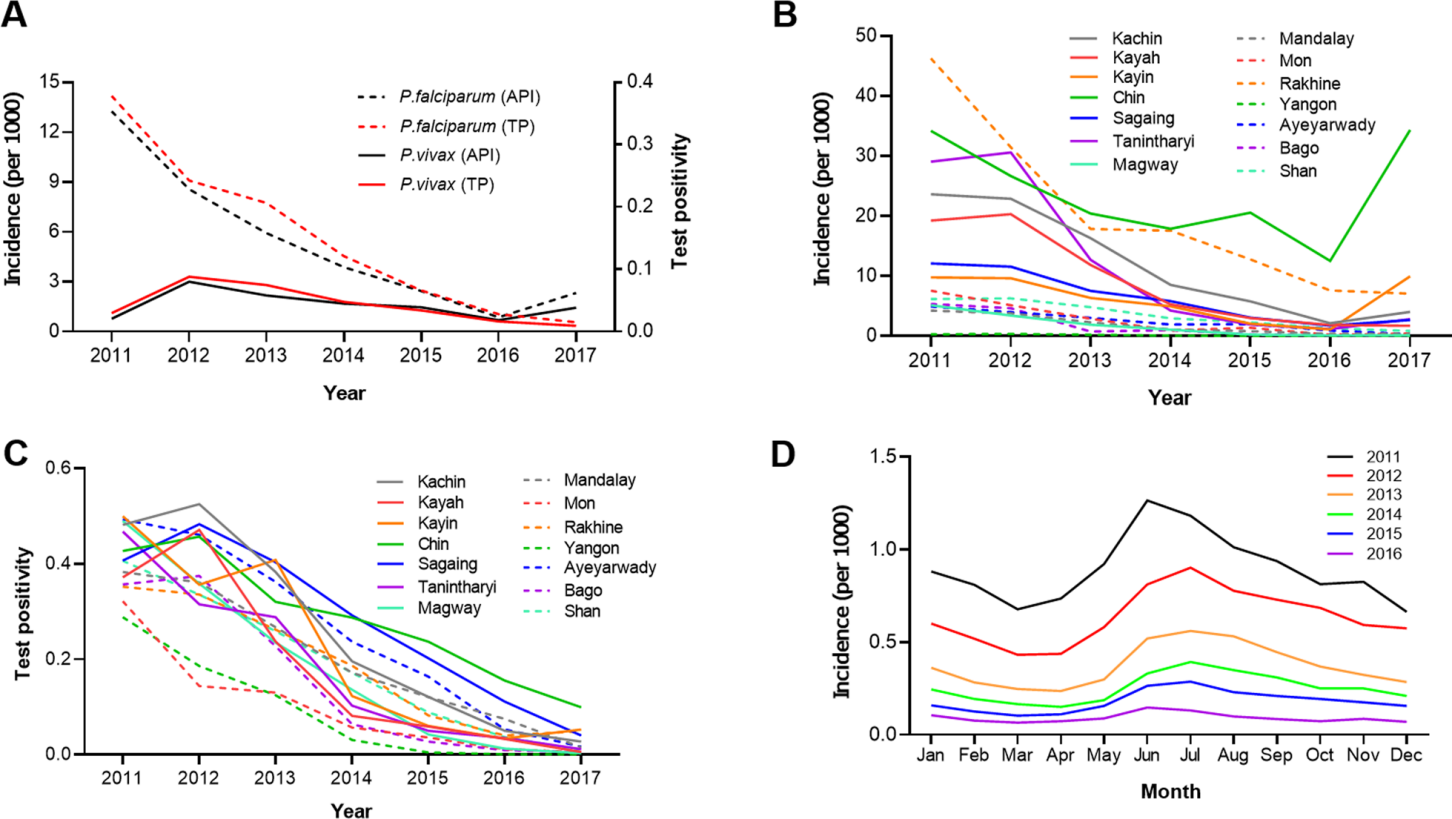
**A** Dynamics of malaria incidence (number of cases per 1000 population) and test positivity among Myanmar residents from 2011 to 2017. **B** State/region wide malaria incidence rate from 2011 to 2017. **C** Test positivity of malaria at state/region level from 2011 to 2017. **D** Monthly incidence of malaria in Myanmar from 2011 to 2016. *API* Annual parasite incidence; *TP* Test positivity

**Fig. 2:**
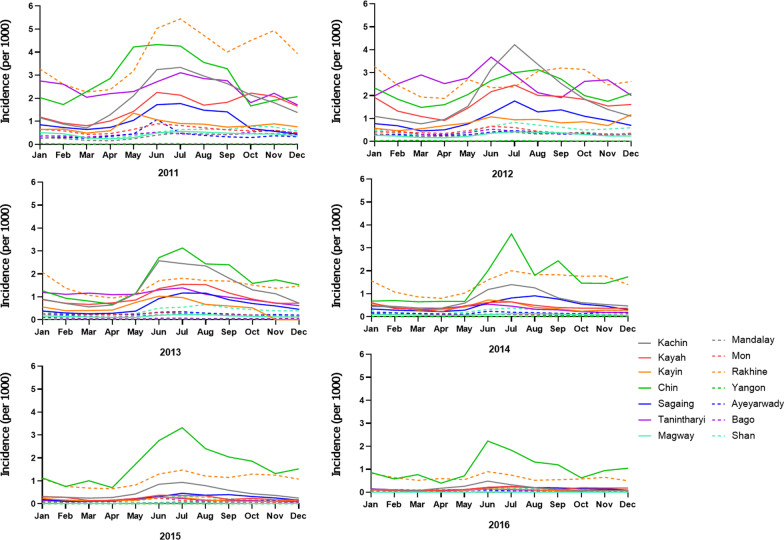
Monthly incidence of malaria at state/region level in Myanmar from 2011 to 2016

From 2011 to 2017, a total of 1,426,737 malaria cases were reported in Myanmar. Malaria API and test positivity in Myanmar showed an overall decline, especially since 2012 (Fig. 1A). From 2012 to 2016, *P. falciparum* cases had a relatively sharp decline, with a 9-fold reduction in API from 8.5 per 1000 in 2012 to 0.9 per 1000 in 2016. Test positivity was similarly reduced from 32.6% in 2012 to 0.7% in 2016. In contrast, the decrease in *P. vivax* cases was gradual, with a 10-fold reduction in API from 3.0 per 1000 in 2012 to 0.3 per 1000 in 2016 and test positivity decreasing from 8.8% in 2012 to 1.6% in 2016. Compared to 2016, both *P. falciparum* and *P. vivax* API experienced a rebound in 2017, although test positivity for both species continually declined (Fig. 1A). The annual API and test positivity in individual regions and states were generally consistent with the overall trend (Fig. 1B, C), while the incidence in the western border state Chin and southern border state Kayin showed a significant upward trend in 2016‒2017, responsible for the overall increase of malaria API in Myanmar in 2016‒2017 (Fig. 1B). Monthly incidence was generally high in the rainy season (May‒September), with the peak occurring in June or July each year (Fig. 1D). Some areas have a second peak in the cool/dry season (November–January, especially Rakhine and Chin states) (Fig. 2). It is noteworthy that Chin, Rakhine, and Kachin states ranked as the top malaria burden states in all years except 2012 (Fig. 2).

### Township-level and region-level patterns of malaria incidence

**Fig. 3:**
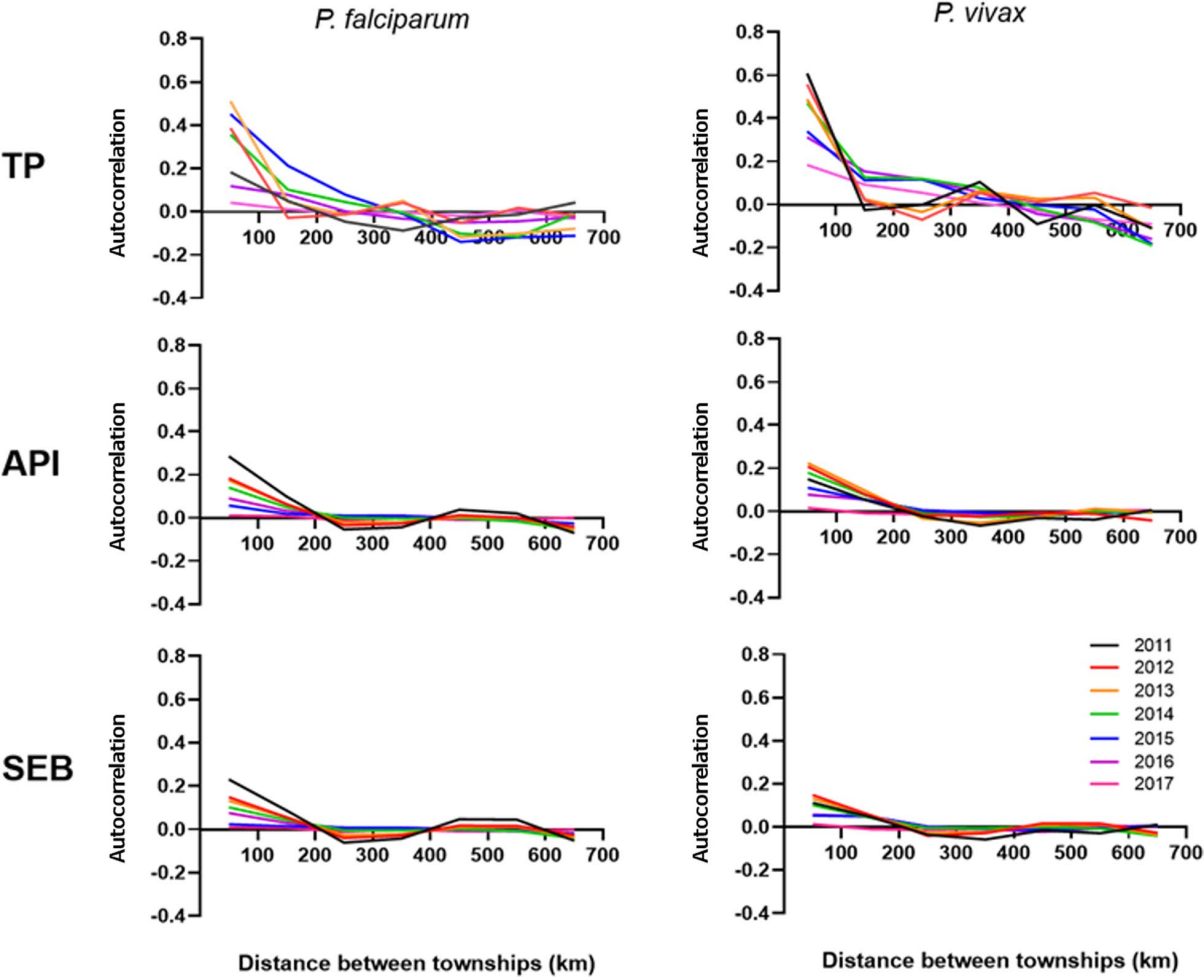
Yearly spatial correlograms of *P. falciparum* malaria and *P. vivax* malaria. *TP* Test positivity; *API* annual parasite incidence; *SEB* smoothed empirical Bayesian rates (API that has been smoothed using the SEB approach)

At the township level, *P. falciparum* and *P. vivax* malaria exhibited similar spatial patterns. Correlograms of township *P. falciparum* and *P. vivax* malaria incidence showed significant spatial autocorrelation up to 100 km (Fig. 3), indicating that malaria incidence in Myanmar was not randomly distributed but rather occurred as clusters among adjacent townships. Also, in the autocorrelation analysis of the monthly incidence from 2011 to 2016 (based on region-level data), we found that the incidence was positively correlated within the range of 100 km except in 2014, further corroborating the above result (Additional file 1: Figs. S1, S2, S3).

### Spatial distribution and cluster of *P. falciparum* and *P. vivax* incidence

**Fig. 4:**
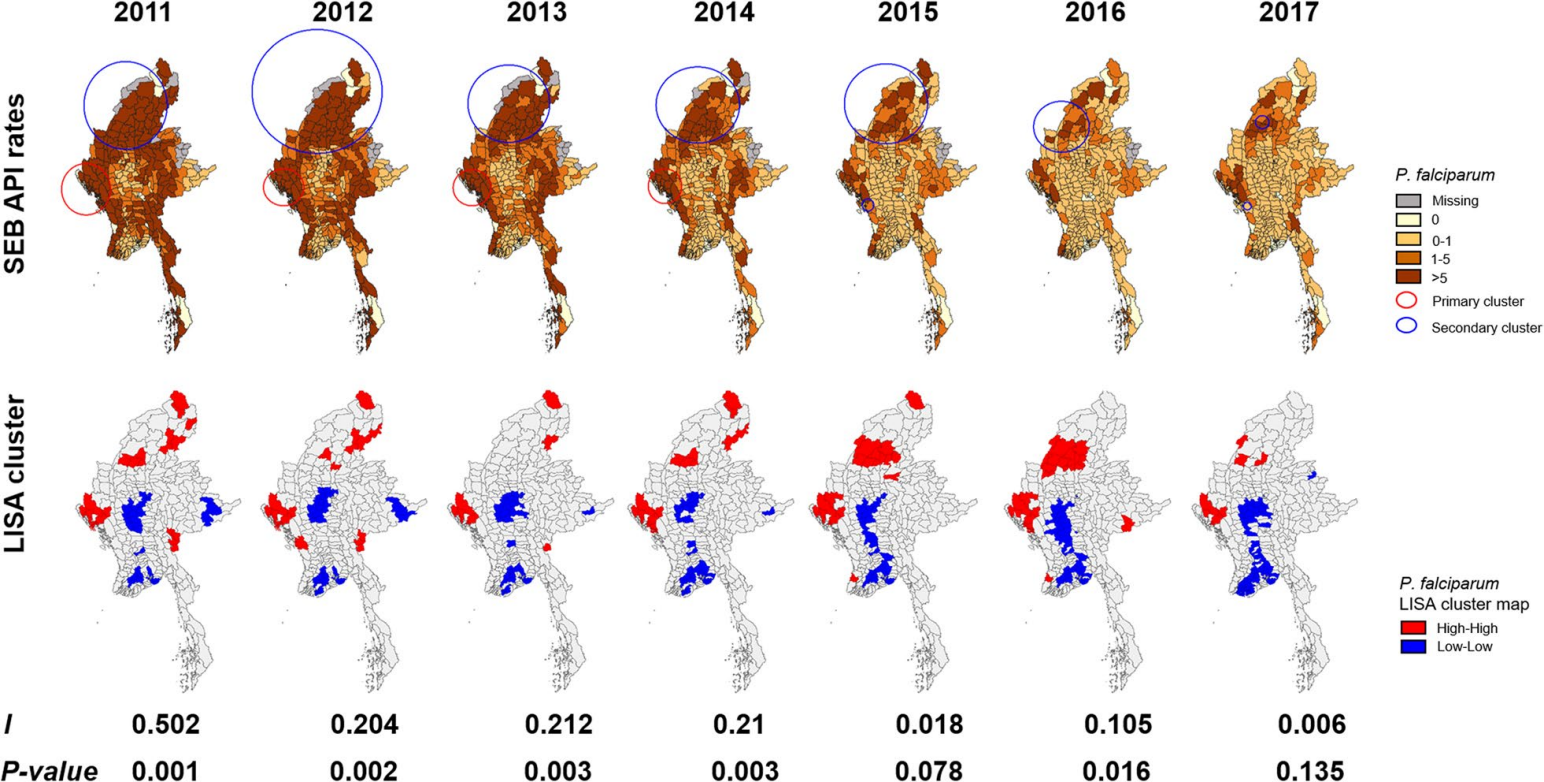
Maps of estimated *P. falciparum* malaria incidence (top row) and spatial autocorrelation. Likely clusters are indicated with red and blue circles. Statistical clusters of high and low numbers of cases (from LISA statistics) are indicated in the lower row. The Myanmar map is generated based on the latest Myanmar Information Management Unit (MIMU) shapefiles and administrative unit codes version 9.3 (http://geonode.themimu.info/)

Analysis of the smoothed APIs showed that areas with higher incidence of *P. falciparum* malaria were concentrated along international borders (Fig. 4). Despite the substantial reduction in *P. falciparum* malaria incidence over the study period, the distribution pattern remained. The number of townships reporting no *P. falciparum* malaria increased from 58 (17.6%) in 2011 to 69 (20.9%) in 2016, but decreased to 52 (15.8%) in 2017 (Fig. 4; Table 1). The number of townships with an API of 0‒1 per 1000 increased over the study period. In 2016 and 2017, the number of townships with *P. falciparum* API of 0‒1 per 1000 was 100 (30.3%) and 118 (35.8%), respectively (Fig. 4; Table 1). The number of townships with an API of > 5 per 1000 sharply decreased from 231 (70.0%) in 2011 to 71 (21.5%) in 2017 (Fig. 4; Table 1).

**Table 1:** Number of townships by *P. falciparum* or *P. vivax* incidence rate groups for each year. Incidence is per 1000 population per year using SEB smoothed rates

Incidence rate	2011	2012	2013	2014	2015	2016	2017
*P. falciparum*							
0	58	58	58	62	61	69	52
0‒1	17	21	19	20	61	100	118
1‒5	24	31	48	84	103	100	89
> 5	231	220	205	164	105	61	71
*P. vivax*							
0	69	60	62	60	59	65	44
0‒1	97	26	24	46	89	109	130
1‒5	106	61	82	95	92	108	94
> 5	58	183	162	129	90	48	62

From the LISA statistics, clustered high API townships (red) were confined to the northern and western parts of Chin State, Kachin State, and Saging Region, while the clustered low API townships (blue) were gathered in the central and southern parts. The spatial autocorrelation (Global Moran’s *I*) ranked between 0.105 and 0.502 (*P* < 0.016) during 2011‒2014 and 2016, revealing a significant and positive spatial autocorrelation in Myanmar (Fig. 4, also Additional file 1: Figs. S4, S5). Over the seven years, the largest malaria clusters were concentrated in western areas of Myanmar. The number of townships covered by the primary clusters decreased from 19 in 2011 to 1 in 2017. Secondary clusters were characterized in the northwest areas (Fig. 4; Table 2).

**Table 2:** The clusters of *P. falciparum* cases detected using the purely spatial clustering in 2011–2017, Myanmar

Year	Cluster type	Cluster areas (*n*)	Observed	Expected	RR	Radius (km)	LLR	*P* value
2011	A	19	82,306	16,321.67	6.07	193.69	73,093.61	0.000
	B	37	96,774	30,464.18	3.86	301.82	51,754.42	0.000
2012	A	17	50,982	9558.10	6.42	134.74	47,658.75	0.000
	B	51	74,841	26,736.30	3.55	458.53	34,438.70	0.000
2013	A	16	40,162	6330.15	7.98	128.30	44,093.98	0.000
	B	31	40,085	11,314.10	4.32	274.45	24,683.83	0.000
2014	A	15	33,491	3736.28	12.73	126.67	48,613.24	0.000
	B	31	27,116	6868.33	4.98	274.45	19,255.70	0.000
2015	A	1	6972	106.48	74.80	0	22,736.04	0.000
	B	31	14,535	3633.68	5.07	274.45	10,492.98	0.000
	B	1	4288	356.01	12.98	0	6883.72	0.000
	B	2	1278	196.47	6.64	44.97	1322.27	0.000
2016	A	1	4135	50.73	96.54	0	14,448.37	0.000
	B	21	6987	1411.46	6.38	184.96	6277.35	0.000
	B	1	669	62.58	10.94	0	985.75	0.000
2017	A	1	13,016	42.98	407.00	0	63,212.89	0.000
	B	3	6001	347.99	19.42	59.06	11,764.61	0.000
	B	2	3497	180.67	20.71	44.97	7156.41	0.000

Type, A: type of most likely cluster and B: second most likely cluster; *n*: the cluster number of township was identified by Kulldorff’s spatial scan; RR: relative risk; LLR: log likelihood ratio

**Fig. 5:**
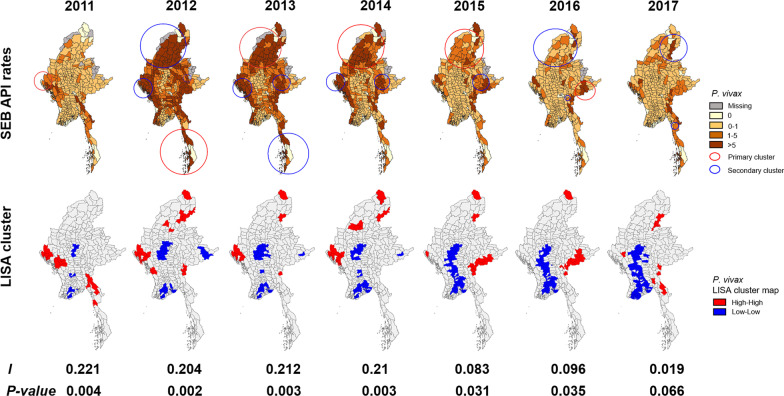
Maps of estimated *P. vivax* malaria incidence (top row) and spatial autocorrelation. Likely clusters are indicated with red and blue circles. Statistical clusters of high and low numbers of cases (from LISA statistics) are indicated in the lower row. Myanmar map is generated based on the latest Myanmar Information Management Unit (MIMU) shapefiles and administrative unit codes version 9.3 (http://geonode.themimu.info/)

The *P. vivax* high API areas were also concentrated along international borders, and the number declined from 2012 to 2017. The number of townships reporting no *P. vivax* malaria cases decreased from 69 (20.9%) in 2011 to 44 (13.3%) in 2017 (Fig. 5; Table 1). The number of townships with smoothed *P. vivax* API of 0‒1 per 1000 increased from 97 (29.4%) to 130 (39.4%) (Fig. 5; Table 1). In 2016 and 2017, the number of townships with a *P. vivax* API of > 5 per 1000 was 48 (14.6%) and 62 (18.8%), respectively (Fig. 5; Table 1). The clustered high API townships (red) were confined to the western, northern, and southeastern parts of Chin, Kachin, and Kayah states. For *P. falciparum*, clustered low API townships (blue) were gathered in central and southern parts. The spatial autocorrelation ranked between 0.083 and 0.221 (*P* < 0.035) during 2011*‒*2016, revealing a significant and positive spatial autocorrelation in Myanmar during this period. In 2017, there was no significantly spatial autocorrelation (Fig. 5, also Additional file 1: Figs. S6, S7). Unlike *P. falciparum* malaria, primary clusters of *P. vivax* malaria changed over the seven years. The number of townships covered by the primary clusters decreased from 37 in 2013 to 1 in 2017. Secondary clusters were characterized in the northern areas in the last two years (Fig. 5; Table 3).

**Table 3:** The clusters of *P. vivax* cases detected using the purely spatial clustering in 2011–2017, Myanmar

Year	Cluster type	Cluster area (*n*)	Observed	Expected	RR	Radius (km)	LLR	*P* value
2011	A	11	13,238	786.80	27.86	126.67	27,794.72	0.000
2012	A	11	17,224	3070.77	6.67	333.23	16,733.83	0.000
	B	17	12,349	3481.29	3.94	134.74	7226.23	0.000
	B	37	16,873	6907.02	2.77	301.82	5709.88	0.000
	B	4	2100	396.65	5.39	51.56	1812.46	0.000
2013	A	37	14,156	4660.00	3.64	301.82	7062.14	0.000
	B	16	7584	2284.14	3.64	134.65	4042.55	0.000
	B	10	6136	1745.13	3.79	296.74	3487.38	0.000
	B	11	3908	829.83	4.96	111.17	3055.90	0.000
2014	A	36	10,291	3027.27	4.20	298.48	6070.39	0.000
	B	12	3924	580.24	7.37	111.96	4298.66	0.000
	B	11	4733	1003.25	5.20	126.67	3791.77	0.000
2015	A	38	7751	2097.52	4.70	261.96	5132.20	0.000
	B	12	3207	402.04	8.86	111.96	3999.60	0.000
	B	1	1297	54.95	24.68	0	2885.72	0.000
2016	A	8	2250	209.46	12.42	120.51	3446.12	0.000
	B	1	797	29.49	28.46	0	1879.63	0.000
	B	31	3227	1006.40	3.80	274.45	1721.89	0.000
	B	1	529	25.08	21.81	0	1117.38	0.000
	B	5	286	39.16	7.42	35.22	323.86	0.000
2017	A	1	5389	22.38	289.26	0	24,663.74	0.000
	B	1	2781	27.17	111.98	0	10,239.71	0.000
	B	9	4107	434.45	10.69	181.89	5775.22	0.000
	B	3	3559	437.37	9.03	49.81	4499.05	0.000

Type, A: type of most likely cluster and B: second most likely cluster; *n*: the cluster number of township was identified by Kulldorff’s spatial scan; RR: relative risk; LLR: log likelihood ratio

### Temporal clustering

The temporal clustering of townships per year was analyzed to identify periods with a higher than expected number of cases, controlling for the population in the respective administrative units. For each stratum, a temporal cluster was identified if there was the same seasonal pattern of high malaria incidence between states/regions in a given year. The temporal cluster decreased in length during the study period (June‒November in 2011 to June‒July in 2017) (Table 4). The temporal cluster analysis also showed that malaria incidence was concentrated in the rainy months, ranging from June to November. The range of months with higher temporal clustering varied slightly from year to year. In 2016, malaria risk was higher in June‒July. From January 2011 to October 2013, there was a temporal cluster of malaria cases in the study area (LLR = 207,689.62, *P* = 0.001). A total of 1,001,309 malaria cases occurred during the same period, and the risk of malaria infection was 3.27 times that of other periods (Table 4).

**Table 4:** The clusters of malaria cases detected using the purely temporal clustering in Myanmar 2011–2016

Year	Cluster time frame	Observed	Expected	RR	LLR	*P* value
2011	June 2011‒November 2011	263,791	217,413.54	1.54	10,001.22	0.001
2012	June 2012‒October 2012	191,492	156,514.83	1.46	6616.78	0.001
2013	June 2013‒September 2013	103,288	74,790.01	1.71	7765.72	0.001
2014	June 2014‒September 2014	69,368	50,870.66	1.67	4818.10	0.001
2015	June 2015‒September 2015	47,765	34,955.17	1.67	3361.85	0.001
2016	June 2016‒July 2016	13,924	9154.33	1.70	1327.50	0.001
2011‒2016	January 2011‒October 2013	1,001,309	634,364.34	3.27	207,689.62	0.001

RR: relative risk; LLR: log likelihood ratio

### Spatio-temporal clusters of malaria

The space-time Poisson model showed two spatio-temporal malaria clusters from 2011 to 2016 (Table 5). The primary cluster was detected from January 2011 to December 2013. The primary cluster covered two states (Sagaing and Kachin, Fig. 5) and persisted from 2011 to 2016.

**Table 5:** Spatial-temporal high risk clusters of malaria cases detected using space–time Poisson model from 2011 to 2016

Cluster	Location	Start date	End date	LLR	RR	Radius	*P* value
1*	2	Jan 1, 2011	Dec 31, 2013	271,025.27	8.04	222.25	0.000
2	2	Jan 1,2011	Nov 30, 2013	118,977.23	3.32	230.75	0.000

*Primary cluster; RR: relative risk; LLR: log likelihood ratio

### Relationships between reported malaria cases and environmental variables

**Fig. 6:**
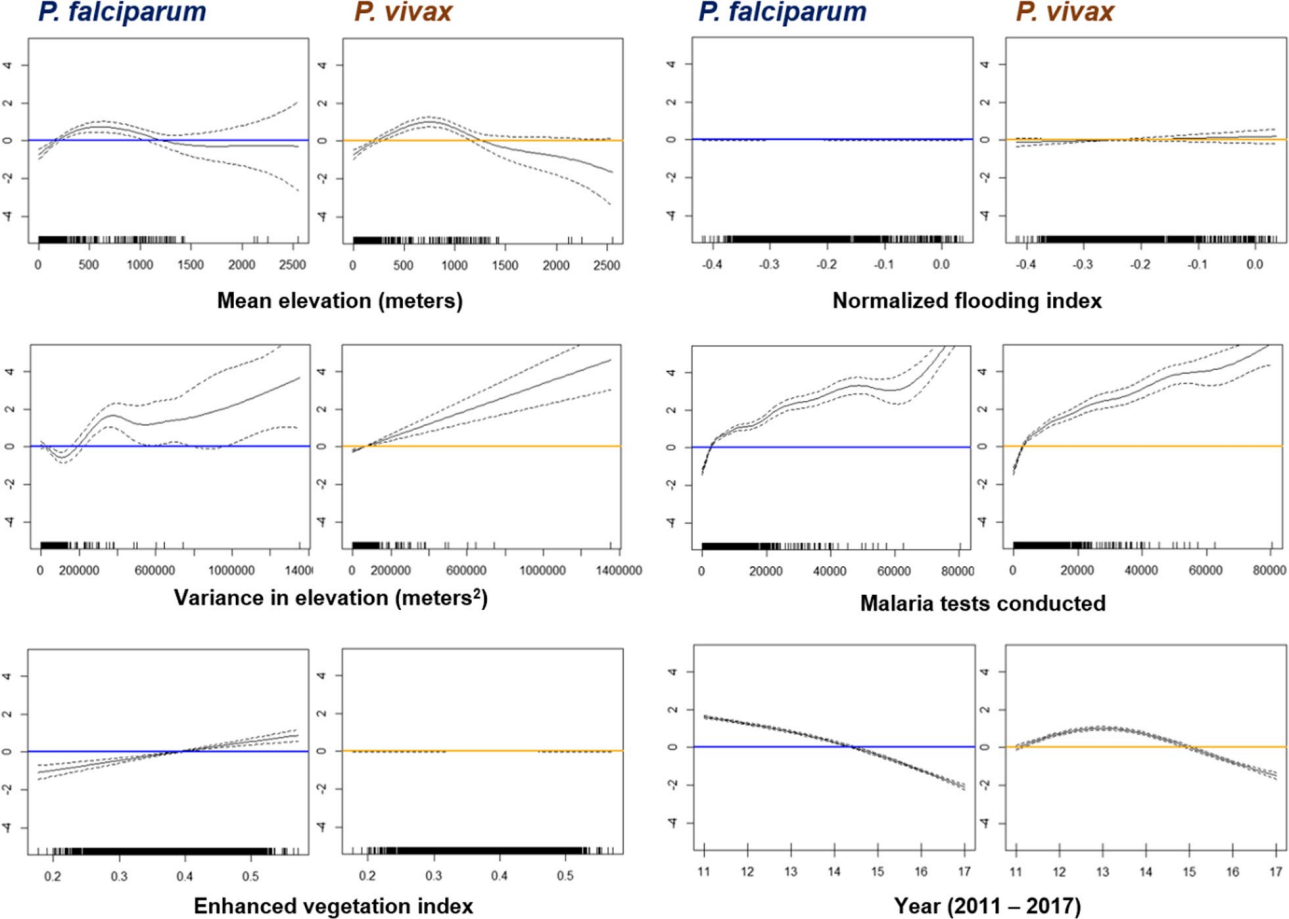
Results from the generalized additive model for environmental predictors of *P. falciparum* and *P. vivax* cases at the township level. Where a spline and its confidence intervals fall above zero (blue and yellow line in figures), there is a positive association, and where they fall below zero (blue and yellow line in figures), there is a negative association. The x-axis gives the value for the variable in question

Potential associations between variables in our negative binomial regression and malaria cases are assessed through plots of the spline functions for each variable (Fig. 6). Annual reported *P. falciparum* cases were highest in townships with a mean elevation of approximately 500–600 m (Fig. 6) and high variance (i.e., townships with both high and low elevations). There was an association between reported vivax malaria cases and elevation as well, but the peak association was at a slightly higher elevation (700–800 m). There was an apparent linear relationship between mean EVI and annual *P. falciparum* cases (Fig. 6), but not with *P. vivax* cases at the township level. There was a decrease in overall *P. falciparum* and *P. vivax* cases over the years, though the decrease was steeper for falciparum malaria and curvilinear for vivax malaria [first increasing until 2013 and then decreasing each year after (Fig. 6)].

## Discussion

Our findings showed that malaria incidence reduced in Myanmar from 2011 to 2017. There was significant spatial autocorrelation in malaria cases and test positivity across the nation. Areas with higher malaria incidence were concentrated along international borders. Primary clusters of *P. falciparum* malaria persisted in western townships, while clusters of *P. vivax* shifted geographically over the study period. Malaria cases were higher in townships with a mean elevation of 500‒600 m and falciparum cases were associated with higher levels of vegetation.

In this study, we used an array of analytic approaches to explore spatial and temporal patterns of malaria API and test positivity based on *P. falciparum* and *P. vivax* data aggregated at the township or state/region level from 2011 to 2017 in Myanmar. The NMCP categorizes geographic locations based on reported case incidence levels (API), with three overarching categories that have corresponding public health approaches: less than 1 per 1000 people per year (pre-elimination), 1 to less than 10 per 1000 people per year (moderate transmission), and above 10 per 1000 people per year (high transmission) [25]. Our findings showed that more than 50% of townships reached malaria pre-elimination by 2017.

The reported *P. falciparum* API maintained a continuous decline throughout the study period, which may be mainly related to increased access to diagnoses and treatment with artemisinin-based combination therapies (ACT) [26]. However, the reported vivax malaria API showed a more gradual decline, probably associated with the intrinsic biological features of *P. vivax*, including relapses induced by hypnozoites [27], missed diagnosis because of the low density of infection, lower accuracy from widely used rapid diagnostic tests [28], the early production of gametocytes favoring continuous transmission, and the high proportion of asymptomatic infections [29].

Some patterns with vivax malaria may be related to the increased roll-out of rapid diagnostic tests that detect both falciparum and vivax malaria (the first rapid diagnostic tests were only capable of detecting falciparum malaria). This roll-out corresponds to the initial increase in vivax malaria cases up to 2013 when they began to decrease (apparent in Fig. 1) and could be related to an increased capacity for diagnosing vivax malaria. A few studies have also shown that vivax malaria relapses following the treatment of falciparum malaria [30, 31]. It is, therefore, possible that widespread increased treatment of falciparum malaria partially drove the short increase of reported vivax malaria cases prior to their decline in 2013. Complete cure of vivax malaria is hindered by the risk of hemolysis in glucose-6-phosphate dehydrogenase deficient (G6PDd) individuals post primaquine and other 8-aminoquinoline antimalarials. This region has a high prevalence of G6PD deficiency along the China-Myanmar border (16.9%) and Thailand–Myanmar border (13.7%) [32, 33], as well as in western and central Myanmar (10% and 6.8%, respectively) [34, 35]. The need for primaquine to radically cure vivax malaria, coupled with the risk of treating G6PDd patients and the difficulties in diagnosing G6PD deficiency in field settings, remains a challenge for adequately addressing this malaria species.

The apparent declines in the API and test positivity of *P. falciparum* and *P. vivax* malaria from 2011 to 2017 in Myanmar may be illustrative of the impact of combined efforts by governmental and non-governmental organizations to eliminate malaria in the nation [36, 37]. Previous studies showed significant associations between declining reported cases and the following factors: long-lasting insecticidal net/insecticide-treated bed net distribution, indoor residual spraying, the concentration of village health workers, amount of health worker training, development of volunteers, socioeconomic status, and improved ACT availability [36, 38–43].

Our analyses also showed malaria to peak and cluster temporally from approximately May and into November. This corresponds with the rainy season in Myanmar, and the pattern has been well-described in several other studies [14, 44]. A second peak was apparent in some high burden states/regions (especially Rakhine and Chin states). This pattern has likewise been described in other locations but has not been fully explained. It may correspond to differences in human-mosquito exposure that are related to seasonal agriculture-the second peak often corresponds with rice harvesting season [45, 46], with changes in mosquito abundance [47], or other factors such as decreased mosquito net use after the rains have ceased [48].

Malaria exhibited significant spatial clustering during the study period as well. We found clustering of *P. falciparum* in the western and northern parts of Chin State, Kachin State, and Sagaing Region. From the Poisson model, we found that primary and secondary clusters of *P. falciparum* also persisted in the northern and western regions of Myanmar. Although the sizes and locations of *P. falciparum* clusters became gradually smaller over time, the cluster locations were relatively stable in the western and northern regions [36, 49]. A recent study found *P. falciparum* was the predominant species accounting for more than 80% of all infections in Paletwa Township of Chin State [46]. Conversely, primary and secondary clusters of *P. vivax* malaria changed over the seven-year study period. The maps of incidence and test positivity demonstrate that high-burden areas of *P. vivax* malaria tend to migrate west to east. In the last three years of the study period, *P. vivax* clustered in eastern Kachin State bordering China and southern Shan State and Kayah State bordering Thailand. Previous studies showed *P. vivax* is the predominant species along many of these international borders [29, 50, 51].

Spatial patterns in malaria can be largely driven by environmental factors that vary across landscapes [52, 53]. Forested areas have long been associated with malaria in Southeast Asia [54, 55]. The hilly and mountainous areas along the international borders have had less economic development, at least partially, as a result of long-standing conflict in these areas. Changes in environmental attributes (such as forest cover) and other socioeconomic factors can lead to changes in the burden of malaria [56]. In this study, we did not limit our measures of EVI or NFI to specific months. Instead, we took the mean value for each year of the study period. If there is a positive association in the model, the interpretation would then be that a higher mean annual value of EVI in a given year is associated with a higher number of malaria cases, etc. It is true that most cases occur during the rainy season, however in several locations there are double peaks—with the second peak occurring outside of the normal rainy season. Partially for this reason, and likewise because the model is analyzing data that have been aggregated yearly, we prefer to have environmental measures that include but are not limited to the rainy season.

Our analyses showed that malaria cases were highest in townships with a mid-level elevation (mean elevation of approximately 500‒600 m) and that falciparum cases were associated with high levels of vegetation (measured using EVI). The latter corresponds to the well-known macro associations between forests and falciparum malaria. While malaria elimination efforts have increased in the last decades in Myanmar, deforestation has also increased [57], and both may impact malaria transmission.

This study has a few limitations. The data might not be accurate for some years since the data were mainly derived from government health centers and the program’s village malaria volunteers. With regard to the temporal trends, it is noteworthy that the coverage of health care facilities has improved drastically in Myanmar over the last several years. For example, many community-based health clinics were set up in Kayin State (beginning around 2014 and 2015). The increase in malaria diagnosis and surveillance at first might give an impression of increased cases when in fact it is the result of increased diagnosis. We control for the influence of testing through the test positivity metric and by including the number of tests in our regressions. However, we cannot control for missing data (either through problems with surveillance systems or from a sparsity of clinics in some regions). Likewise, we do not have data on the proportions of tests that were done using RDTs or microscopy and the data are at aggregate levels meaning that we cannot perform detailed analyses of demographic risk factors. Furthermore, since our data and analyses are limited to Myanmar, it is possible that some of the patterns we see are obscured along the edges of the map. It is possible that we are missing hotspots and coldspots of malaria that would be apparent if we had corresponding data from neighboring nations (this is a type of ‘edge effect’).

Another potential limitation is related to the environmental variables included in our models. While we are confident that our model results indicate true associations between environmental variables and either falciparum or vivax malaria cases, other environmental variables could be included in similar analyses. For example, while we chose to use EVI this measure of vegetation would not necessarily differentiate forests from other kinds of vegetation. Since our a priori goal was not to look for associations between forests and malaria, we chose this more inclusive variable instead of measures that would be specific to forests.

Another limitation is the deficiency of monthly data, which were only available at the state/region level from 2011 to 2016. More granular data (i.e., at the village or village tract level) would be superior. Lastly, the coup in 2021 and COVID-19 pandemic (beginning in 2020) have disrupted many malaria control and elimination efforts. From passive case surveillance in Laiza town in Kachin State along the China-Myanmar border, malaria cases declined from 2016 to 2019 but increased rapidly beginning in 2020 [58]. This suggests that local governments or organizations should resume malaria surveillance and implementation of interventions as soon as possible. Meanwhile, the bordering countries should pay attention to the importation of malaria cases.

## Conclusion

This study describes spatial and temporal patterns of malaria incidence across the entire nation of Myanmar over a 7-year period (2011–2017). We describe an overall reduction in both falciparum and vivax malaria, which is likely driven at least partially by enhanced malaria elimination and control efforts during this same period. Deforestation and socioeconomic factors may likewise explain part of this reduction. However, malaria continued to cluster in some locations (falciparum malaria in the northwest, vivax malaria in the northeast), and these clusters could act as reservoirs for rebounds in other regions if vigilance is not maintained. Unfortunately, the SARS-CoV-2 (beginning in 2020) pandemic and the military coup in 2021 have disrupted many of these efforts. To achieve elimination, it will be necessary to resume heightened control and elimination efforts, and likely will require tailored approaches to address the complications of eliminating vivax malaria.

## Supplementary Information

**Additional file 1:** **Figure S1. **Monthly spatial correlograms with test positivity for combined malaria cases/tests*. ***Figure S2. **Monthly spatial correlograms with the API (annual parasite incidence) for combined malaria. API is calculated as the number of cases per 1000 people per year. **Figure S3. **Monthly spatial correlograms with SEB (smoothed empirical Bayesian) API for combined malaria. **Figure S4. ***P. falciparum *malaria distribution and spatial autocorrelation statistic using the testpositivity (TP). **Figure S5. ***P. falciparum *malaria distribution and spatial autocorrelation statistic using the annual parasite incidence (API), calculated as the number of cases per 1000 people per year. **Figure S6. ***P. vivax *malaria distribution and spatial autocorrelation statistic using the test positivity (TP). **Figure S7. ***P. vivax *malaria distribution and spatial autocorrelation statistic using the annual parasite incidence (API) calculated as the number of cases per 1000 people per year.

## Data Availability

All data analyzed for this study are included within the article.

## Abbreviations

ACT: Artemisinin-based combination therapies
API: Annual parasite incidence
GMS: Greater Mekong Subregion
MME: Mekong Malaria Elimination
MODIS: Moderate Resolution Imaging Spectroradiometer
NDVI: Normalized difference vegetative index
EVI: Enhanced vegetation index
NFI: Normalized flooding index
RDT: Rapid diagnostic test
LISA: Local indicator of spatial autocorrelation
NMCP: National malaria control program
G6PD: Glucose-6-phosphate dehydrogenase.
