# Supplementary material for: Spatio-temporal trends of malaria incidence from 2011 to 2017 and environmental predictors of malaria transmission in Myanmar
Source: Infect Dis Poverty. 2023 Jan 28;12:2. doi: 10.1186/s40249-023-01055-6 (PMC9883610; doi:10.1186/s40249-023-01055-6)

**ADDITIONAL FILE**

**Figure S1.** Monthly spatial correlograms with test positivity for combined malaria cases/tests*.*


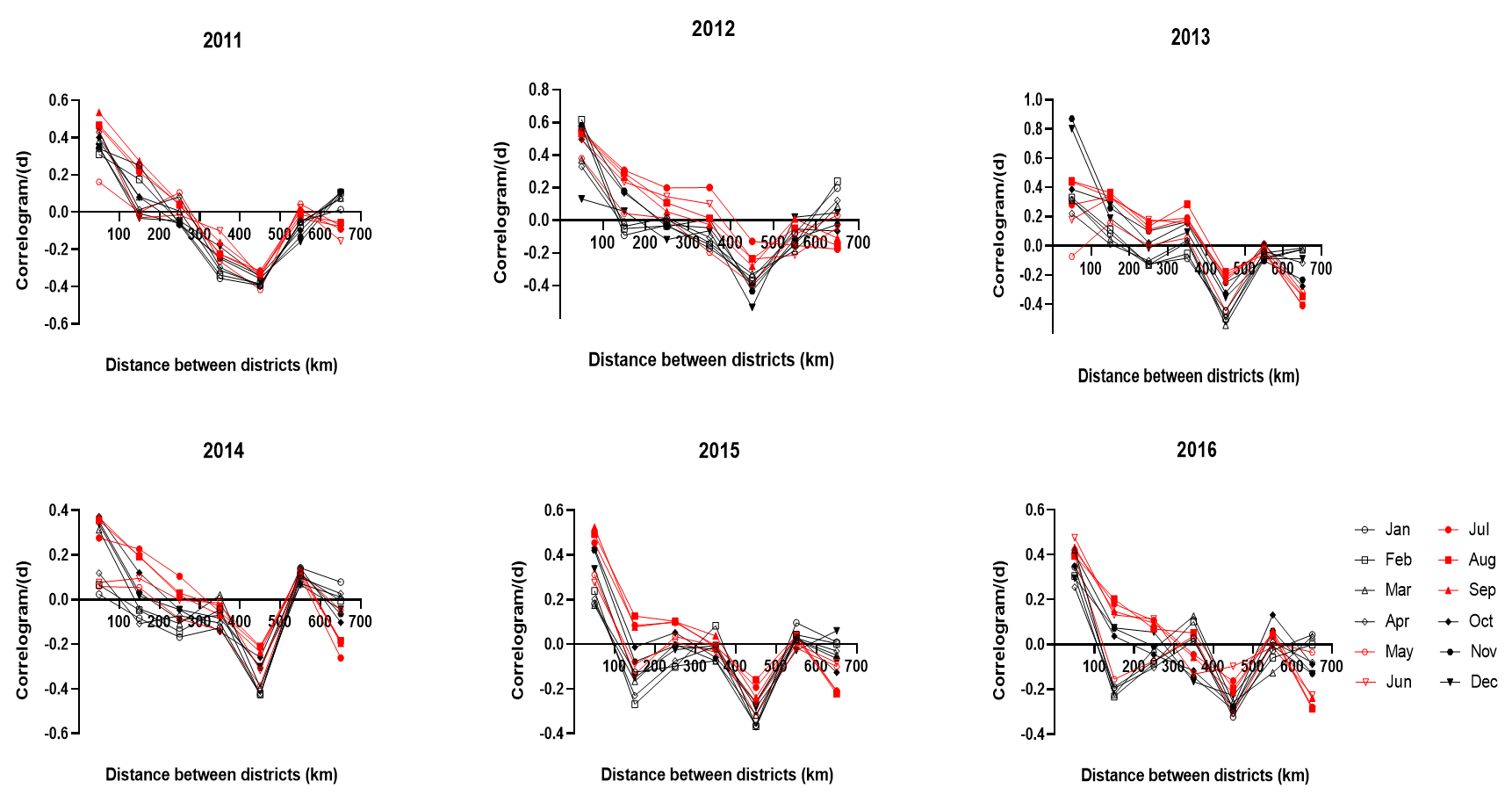


**Figure S2.** Monthly spatial correlograms with the API (annual parasite incidence) for combined malaria*.* API is calculated as the number of cases per 1000 people per year.


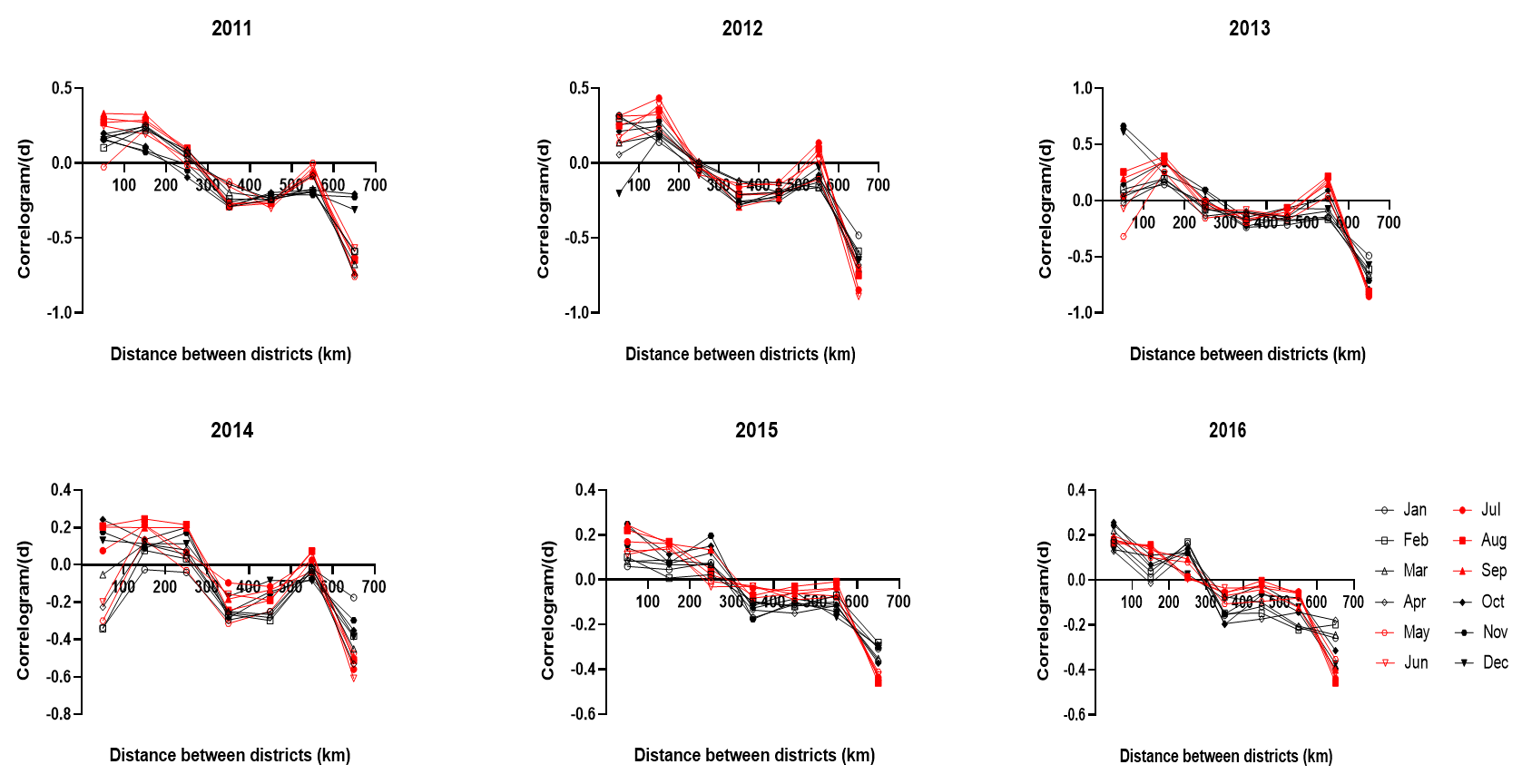


**Figure S3.** Monthly spatial correlograms with SEB (smoothed empirical Bayesian) API for combined malaria.


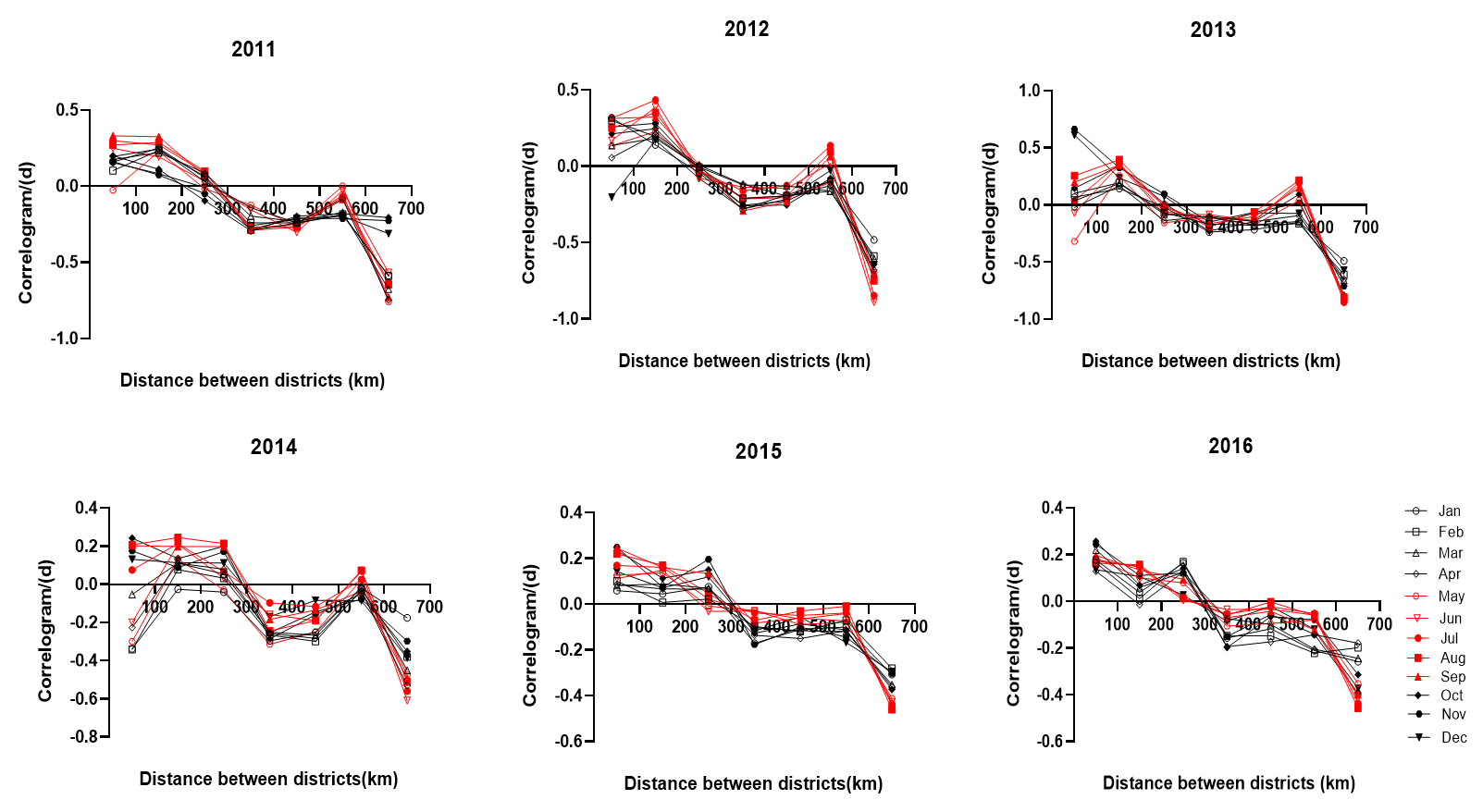


**Figure S4.** *P. falciparum* malaria distribution and spatial autocorrelation statistic using the test positivity (TP).


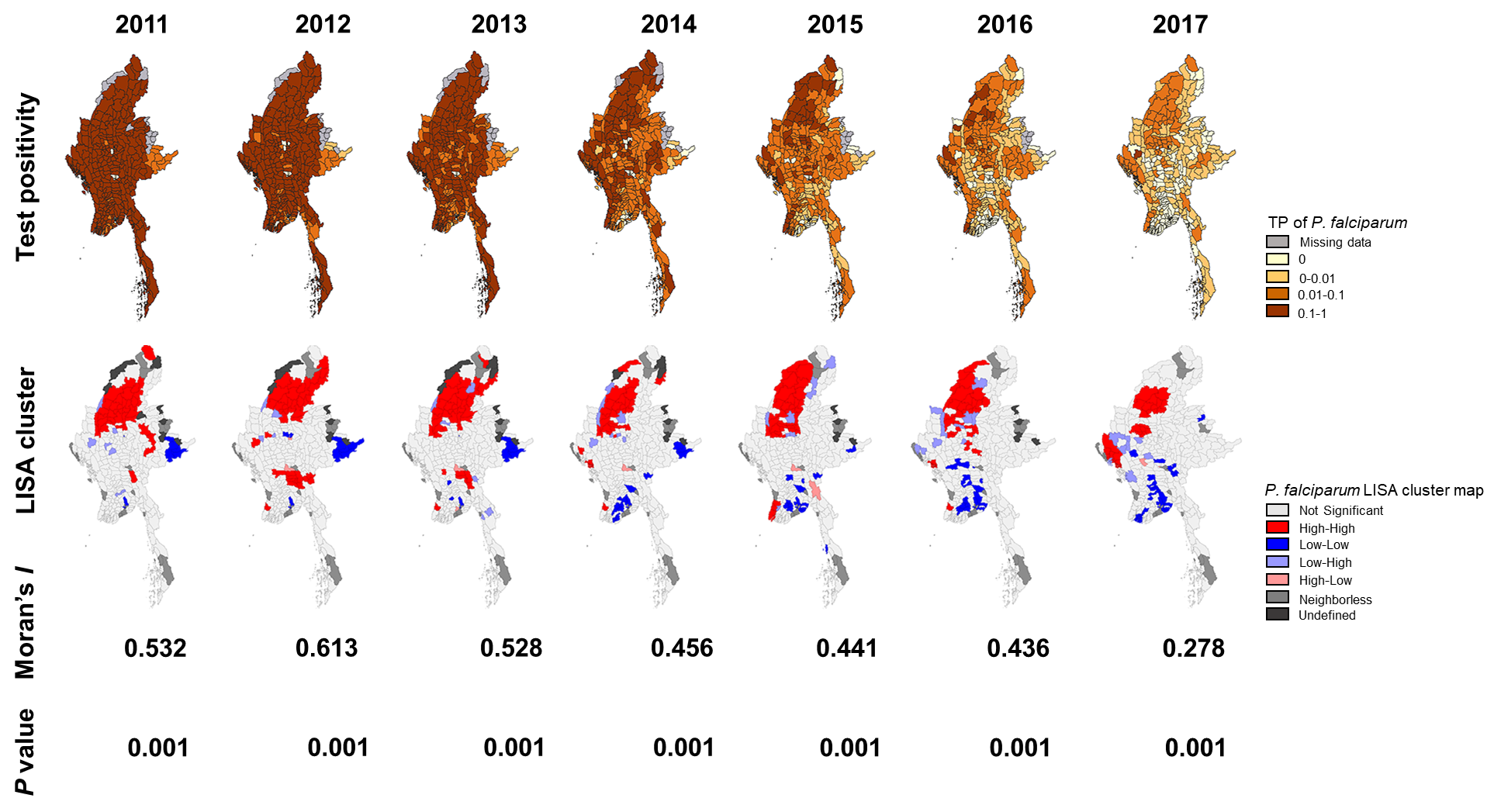


**Figure S5.** *P. falciparum* malaria distribution and spatial autocorrelation statistic using the annual parasite incidence (API), calculated as the number of cases per 1000 people per year.


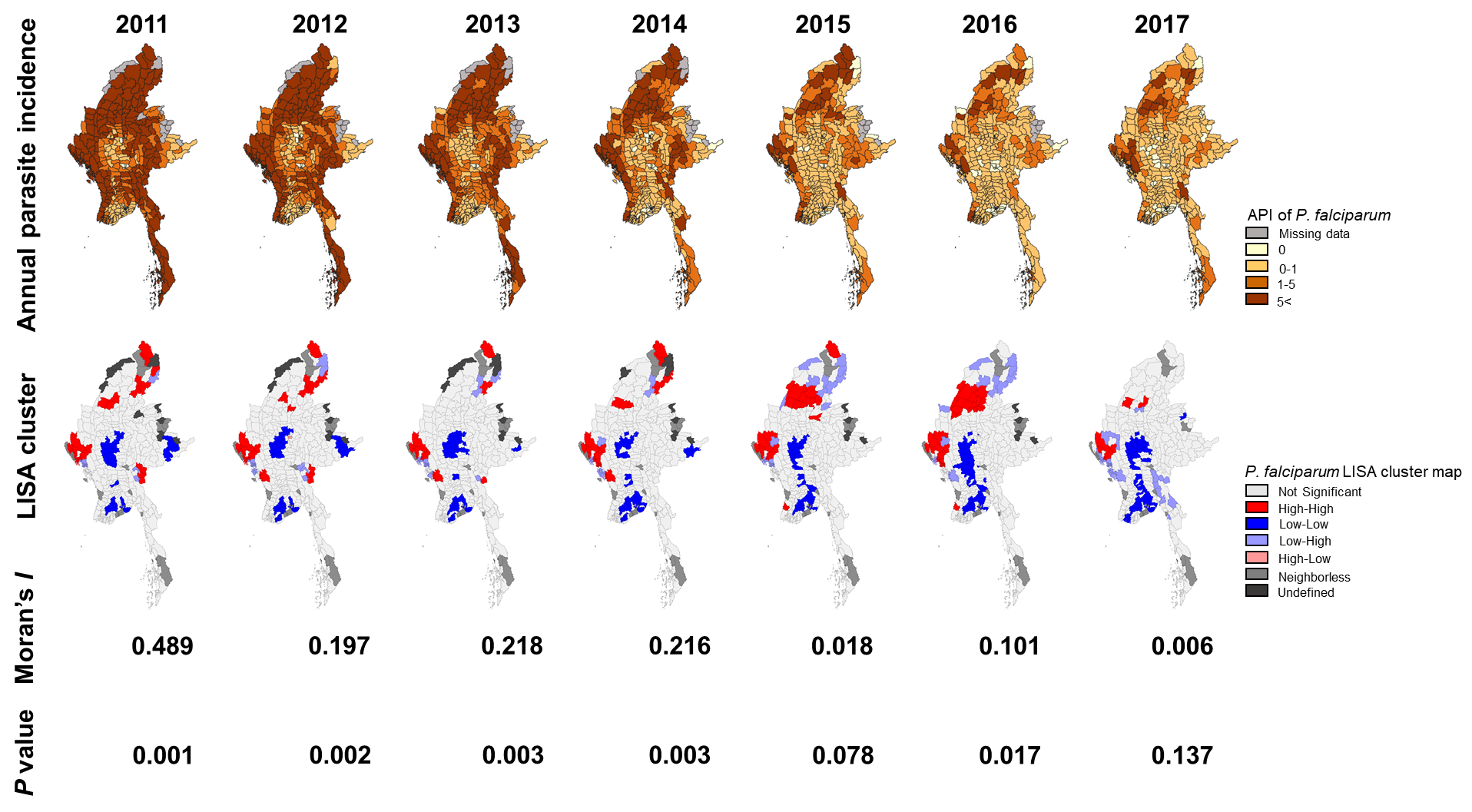


**Figure S6.** *P. vivax* malaria distribution and spatial autocorrelation statistic using the test positivity (TP).


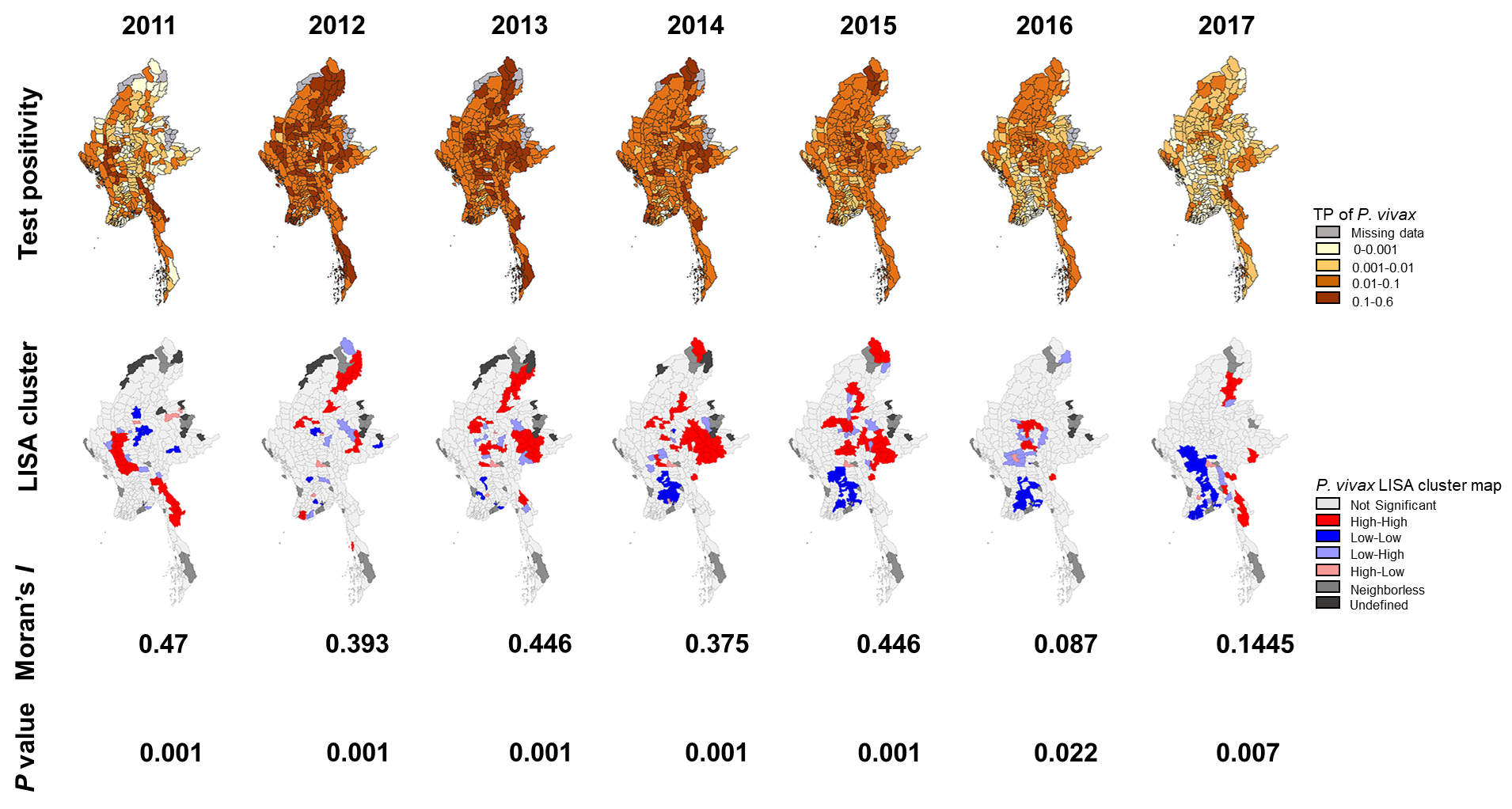


**Figure S7.** *P. vivax* malaria distribution and spatial autocorrelation statistic using the annual parasite incidence (API) calculated as the number of cases per 1000 people per year.


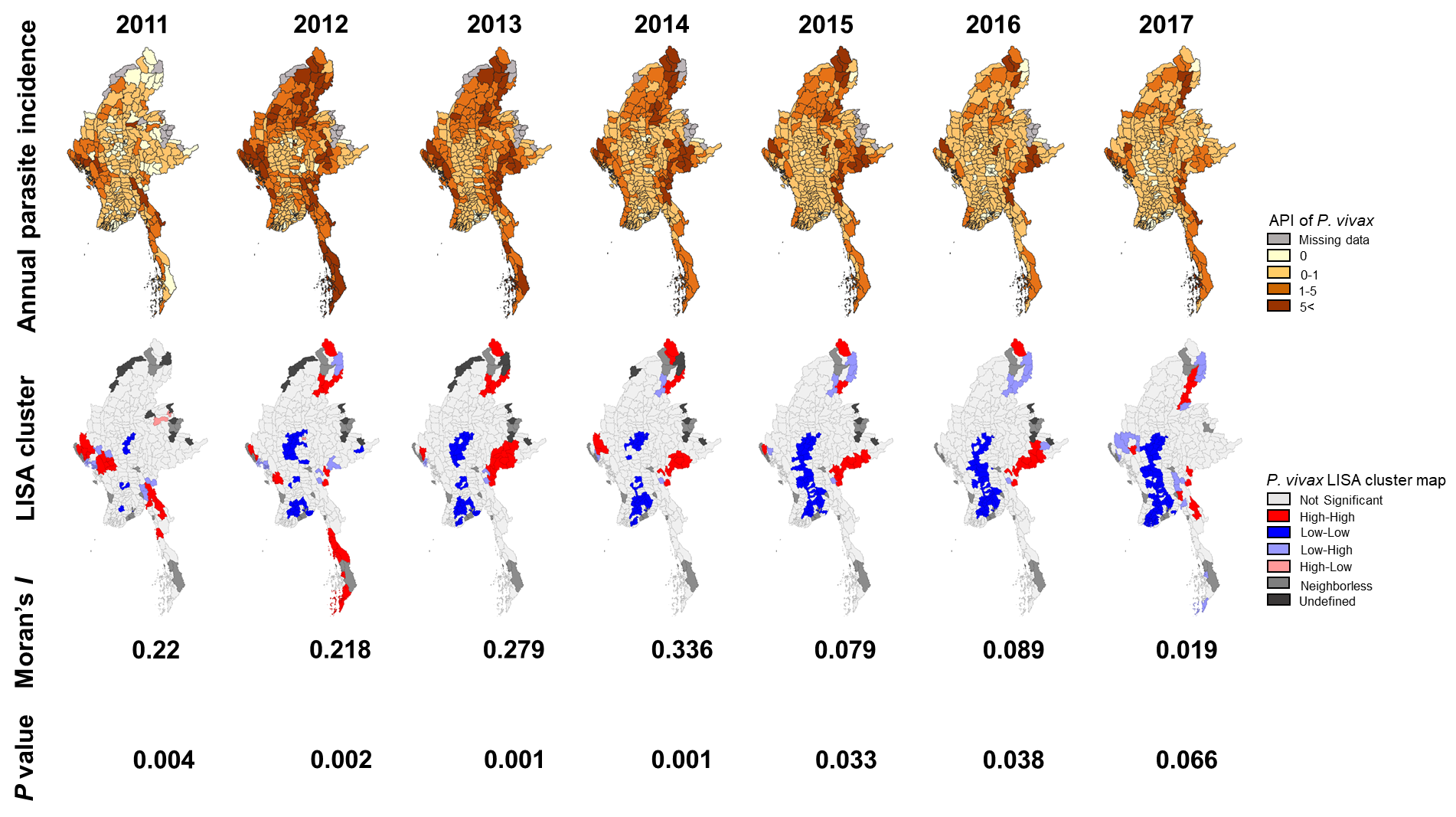

Supplement: Supplementary file 1 — Additional file 1: Figure S1. Monthly spatial correlograms with test positivity for combined malaria cases/tests. Figure S2. Monthly spatial correlograms with the API (annual parasite incidence) for combined malaria. API is calculated as the number of cases per 1000 people per year. Figure S3. Monthly spatial correlograms with SEB (smoothed empirical Bayesian) API for combined malaria. Figure S4. P. falciparum malaria distribution and spatial autocorrelation statistic using the testpositivity (TP). Figure S5. P. falciparum malaria distribution and spatial autocorrelation statistic using the annual parasite incidence (API), calculated as the number of cases per 1000 people per year. Figure S6. P. vivax malaria distribution and spatial autocorrelation statistic using the test positivity (TP). Figure S7. P. vivax malaria distribution and spatial autocorrelation statistic using the annual parasite incidence (API) calculated as the number of cases per 1000 people per year. [file 40249_2023_1055_MOESM1_ESM.docx]
